# An EEG-based machine learning framework for diagnosing acute sleep deprivation

**DOI:** 10.3389/fphys.2025.1668129

**Published:** 2025-11-17

**Authors:** Daya Kumar, Apurva Narayan, Saptharishi Lalgudi Ganesan

**Affiliations:** 1 Department of Computer Science, Western University, London, ON, Canada; 2 International Center for Applied Systems Science for Sustainable Development (ICASSSD), Cambridge, ON, Canada; 3 Department of Paediatrics and Clinical Neurological Sciences, Schulich School of Medicine and Dentistry, Western University, London, ON, Canada; 4 Paediatric Critical Care Medicine, Children’s Hospital - London Health Sciences Centre, London, ON, Canada; 5 Western Institute for Neuroscience, Western University, London, ON, Canada; 6 Children’s Health Research Institute, London Health Sciences Center Research Institute (LHSC-RI), London, ON, Canada

**Keywords:** acute sleep deprivation, electroencephalogram (EEG), ensemble models, feature importance, machine learning

## Abstract

**Study objective:**

Acute sleep deprivation significantly impacts cognitive function, contributes to accidents, and increases the risk of chronic illnesses, underscoring the need for reliable and objective diagnosis. Our work aims to develop a machine learning-based approach to discriminate between EEG recordings from acutely sleep-deprived individuals and those that are well-rested, facilitating the objective detection of acute sleep deprivation and enabling timely intervention to mitigate its adverse effects.

**Methods:**

Sixty-one-channel eyes-open resting-state electroencephalography (EEG) data from a publicly available dataset of 71 participants were analyzed. Following preprocessing, EEG recordings were segmented into contiguous, non-overlapping 20-second epochs. For each epoch, a comprehensive set of features was extracted, including statistical descriptors, spectral measures, functional connectivity indices, and graph-theoretic metrics. Four machine learning classifiers - Light Gradient-Boosting Machine (LightGBM), eXtreme Gradient Boosting (XGBoost), Random Forest (RF), and Support Vector Classifier (SVC) - were trained on these features using nested stratified cross-validation to ensure unbiased performance evaluation. In parallel, three deep learning models-a Convolutional Neural Network (CNN), Long Short-Term Memory network (LSTM), and Transformer-were trained directly on the raw multi-channel EEG time-series data. All models were evaluated under two conditions: (i) without subject-level separation, allowing the same participant to contribute to both training and test sets, and (ii) with subject-level separation, where models were tested exclusively on unseen participants. Model performance was assessed using accuracy, F1-score, and area under the receiver operating characteristic curve (AUC).

**Results:**

Without subject-level separation, CNN achieved the highest accuracy (95.72%), followed by XGBoost (95.42%), LightGBM (94.83%), RF (94.53%), and SVC (85.25%), with the Transformer (77.39%) and LSTM (66.75%) models achieving lower accuracies. Under subject-level separation, RF achieved the highest accuracy (68.23%), followed by XGBoost (66.36%), LightGBM (66.21%), CNN (65.35%), and SVC (65.08%), while the Transformer (63.35%) and LSTM (61.70%) models achieved the lowest accuracies.

**Conclusion:**

This study demonstrates the potential of EEG-based machine learning for detecting acute sleep deprivation, while underscoring the challenges of achieving robust subject-level generalization. Despite reduced accuracy under cross-subject evaluation, these findings support the feasibility of developing scalable, non-invasive tools for sleep deprivation detection using EEG and advanced ML techniques.

## 1 Introduction

Sleep deprivation induces a wide range of neurological and behavioral impairments that significantly undermine cognitive functioning. Sleep deprivation leads to a phenomenon known as ‘localized sleep’, wherein specific brain regions transition into a sleep-like state while the individual remains awake (Vyazovskiy et al., 2011) leading to measurable lapses in cognitive and motor performance (Hung et al., 2013). Moreover, sleep deprivation disrupts the functional connectivity of critical brain networks diminishing communication efficiency across brain regions (Bernardi et al., 2015). Notably, the cognitive effects of sleep deprivation are task-dependent, with executive functions, such as impulse control and visuomotor coordination, exhibiting heightened vulnerability (Bernardi et al., 2015). Moreover, these studies have demonstrated that the increasing occurrence of local neuronal off periods during extended wakefulness correlates with progressive declines in task performance (Hung et al., 2013; Bernardi et al., 2015). Collectively, these findings underscore the essential role of sleep in maintaining cognitive and behavioral stability while highlighting the intricate neural mechanisms through which sleep deprivation compromises brain function.

The real-world implications of these findings are profound, particularly given the high prevalence of acute sleep deprivation in modern society (Gohari et al., 2024). The combination of impaired impulse control, diminished visuomotor coordination and attention lapses significantly increases the likelihood of accidents and human errors (Kayser et al., 2022). These risks are particularly critical in professions requiring sustained attention and precise motor skills, such as healthcare, transportation, and manufacturing. Furthermore, cognitive deficits associated with acute sleep deprivation, including reduced concentration and impaired decision-making (Killgore et al., 2006), negatively affect workplace efficiency and productivity (Brossoit et al., 2019). The link between acute sleep deprivation and diminished impulse control has also been associated with behavioral impulsivity, disinhibition, and increased aggression, contributing to broader implications for mental health and social behavior (Killgore, 2010). Despite the adverse effects of acute sleep deprivation, current detection methodologies primarily rely on self-reported sleep metrics or laboratory-based assessments. While laboratory tests are often resource-intensive and impractical for real-time monitoring (Mitler and Miller, 1996), self-reported metrics are biased or influenced by financial and peer pressure considerations. Therefore, there is a critical need for reliable, objective, and portable tools capable of detecting acute sleep deprivation in real-world settings and mitigating its impact.

Electroencephalography (EEG) has been validated as an effective modality for capturing neural alterations associated with sleep deprivation (Lian et al., 2023). Spectral analysis of EEG signals consistently reveals shifts in brain oscillatory activity that correspond to cognitive and behavioral impairments (Tramonti Fantozzi et al., 2022; Gorgoni et al., 2014; Tassi et al., 2006; Forest and Godbout, 2000). Sleep deprivation is characterized by increased power in low-frequency bands (delta, theta) and reduced power in higher-frequency bands (alpha, beta), particularly in cortical regions implicated in attention and visual processing (Lian et al., 2023; Liu et al., 2025; Hung et al., 2013).

Regional differences in neural activity have been consistently documented. In frontal regions, total sleep deprivation has been shown to lead to a marked loss of functional connectivity in prefrontal cortical areas, characterized by reductions in clustering coefficient in the alpha band and increases in path length in the theta band (Verweij et al., 2014). Additionally, prolonged wakefulness produces a robust increase in frontal low EEG activity (1–7 Hz), reflecting heightened sleep pressure and cortical fatigue (Cajochen et al., 2001). In contrast, in parietal and occipital regions, significant reductions in alpha-band power and increases in delta-band power in the precuneus, inferior parietal lobule, and superior parietal lobule have been reported following 24 h of total sleep deprivation (Lian et al., 2023). Furthermore, theta-band power in centro-parieto-occipital areas increases substantially after prolonged sleep deprivation, with greater elevations correlating with more severe vigilance impairments (Liu et al., 2025). Collectively, these studies highlight the sensitivity of both frontal and posterior cortical regions to sleep loss, demonstrating the effectiveness of EEG in detecting neural alterations relevant to cognitive decline and underscoring its utility for machine learning-based acute sleep deprivation classification.

Machine learning has been widely applied in EEG-based sleep research, with ensemble methods (Mienye and Sun, 2022) and traditional classifiers frequently demonstrating robust performance. Ensemble techniques, such as Random Forest (RF) (Zhao et al., 2022; Monowar et al., 2025), eXtreme Gradient Boosting (XGBoost) (Wang et al., 2024; Monowar et al., 2025), and Light Gradient-Boosting Machine (LightGBM) (Wang et al., 2023; Jain and Ganesan, 2025), have been successfully deployed to model complex, nonlinear patterns in heterogeneous EEG datasets, enhancing generalizability and mitigating overfitting. Support Vector Machines (SVMs) (Kumari et al., 2020) also remain competitive, especially in sleep quality (Wen, 2021) and disorder detection tasks (Falih et al., 2024; Djemal et al., 2025; Bansal et al., 2025; Kim et al., 2025), due to their capacity to accommodate high-dimensional spaces and nonlinear separations.

Deep learning (DL) approaches, including convolutional neural networks (CNNs) (Zhang et al., 2023; Tanci and Hekim, 2025), hybrid CNN–LSTM (Long Short-Term Memory) architectures (Zhuang et al., 2022), and more recently Transformer-based models (Wan et al., 2025), have emerged as powerful tools by learning spatial and temporal representations directly from raw EEG signals. However, studies indicate that, particularly when dataset size is limited, traditional machine learning models, such as SVMs and decision trees, can offer greater robustness and interpretability compared to deep learning approaches, which often require extensive data and computational resources (Rahul et al., 2024).

Despite these advancements, the application of machine learning for the detection of acute sleep deprivation remains underexplored. Existing work in this domain is scarce, with only one notable study to date (Baygin, 2025). Although it achieved high classification accuracy, the study lacked a focus on interpretability, which reduces its relevance for clinical application. Without physiologically grounded explanations, model predictions remain difficult to translate into actionable neurophysiological insights.

The present study aims to address these shortcomings by (i) extracting a comprehensive set of statistical, spectral, functional connectivity, and graph-theoretic features with clear neurophysiological relevance, (ii) implementing a nested stratified cross-validation framework with subject-level separation to derive realistic generalization estimates for unseen individuals. (iii) conducting feature importance analyses to enhance interpretability and lay the groundwork for clinical integration.

## 2 Methods

### 2.1 Dataset

For this study, we leveraged an open-source eyes-open resting-state EEG dataset containing data from 71 healthy young adults (34 females, 37 males), ranging from 17 to 23 years old, with a mean age of 20 ± 1.44 years (Xiang et al., 2024). Participants were excluded if they had a history of psychiatric disorders, anxiety, depressive symptoms, respiratory disturbances during sleep, or recent illness. Sleep quality was assessed prior to participation to confirm normal sleep patterns. A within-subject experimental design was employed, wherein each participant completed two sessions, one during well-rested wakefulness, following a normal sleep cycle and another after acute sleep deprivation.

The order of sessions was counterbalanced to mitigate sequence effects, with an interval of 7 days to 1 month between conditions. To minimize circadian variability, the two sessions for each participant were scheduled within the same fixed timeframe (morning or afternoon), with most participants (81.6%) having less than a 1.5-h difference in session start times between conditions. For the acute sleep deprivation condition, participants remained awake for 24–30 h under continuous monitoring by experimenters. Actigraphy was used to ensure compliance. Both sessions followed an identical testing protocol, beginning with cognitive and behavioral assessments, including the Psychomotor Vigilance Task (PVT) to assess alertness. Resting-state EEG recordings were acquired using a 61-channel system (Brain Products GmbH, Germany) at a sampling rate of 500 Hz. Electrode impedance was maintained below 5 K Ω . EEG data were recorded for five minutes with eyes open. Participants were instructed to fixate on a point, minimize movement, and remain still to ensure high-quality data acquisition. One subject was excluded from data analysis due to incomplete data.

### 2.2 EEG preprocessing

**FIGURE 1:**
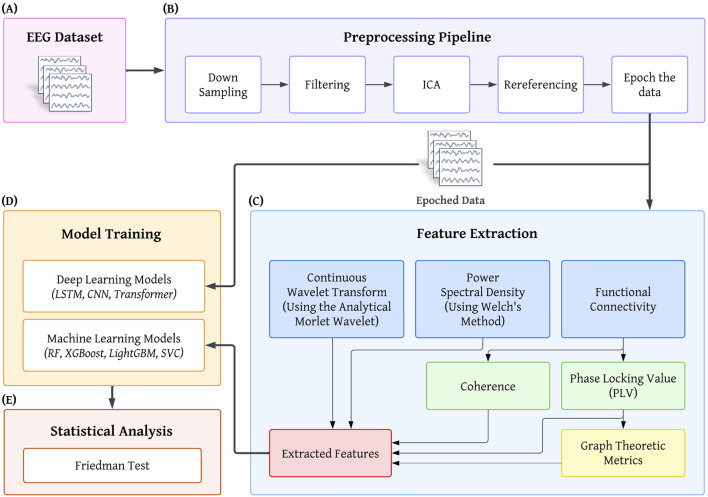
Study Diagram. **(A)** Open-source eyes-open EEG dataset. **(B)** Preprocessing pipeline. ICA refers to Independent Component Analysis. **(C)** Feature Extraction. **(D)** Model Training. Four machine learning models - LightGBM, (Light Gradient-Boosting Machine), XGBoost (eXtreme Gradient Boosting), RF (Random Forest), SVC (Support Vector Classifier), and three deep learning models - LSTM (Long Short-Term Memory), CNN (Convolutional Neural Network), and a Transformer-based model are trained. **(E)** Statistical analysis is done using the Friedman test.

An overview of the EEG preprocessing, feature extraction, and analysis pipeline is presented in Figure 1. Preprocessing was performed in MATLAB (The MathWorks, Inc, 2024) using the EEGLAB toolbox (Delorme and Makeig, 2004). Data from all 61 channels were retained for analysis. The recordings were resampled to 256 Hz and bandpass filtered using a finite impulse response (FIR) filter with cutoff frequencies set at 0.2 Hz and 45 Hz. This filtering range was selected to preserve frequency components relevant to neurophysiological processes while attenuating slow drifts and high-frequency noise.

Artifact correction was conducted using Independent Component Analysis (ICA). To ensure objectivity and reproducibility, components associated with ocular and muscle activity were automatically identified and removed using the ICLabel classifier (Pion-Tonachini et al., 2019), applying a probability threshold of 70% for rejection within each artifact category. Following artifact removal, the data were re-referenced to a common average reference.

Subsequently, the EEG data were segmented into contiguous, non-overlapping 20-second epochs, a duration chosen to balance temporal resolution with the stability of spectral estimates (Möcks and Gasser, 1984; Mohsenvand et al., 2020). Bad epochs were identified using an amplitude-based criterion, whereby any epoch in which the standard deviation of one or more channels exceeded 50 μ V was excluded from further analysis. Following epoch rejection, *18.6*% of epochs were removed across participants. The final dataset included 1,681 epochs, with 858 epochs corresponding to the well-rested wakefulness condition and 823 epochs to the acute sleep deprivation condition.

### 2.3 Feature extraction

To extract meaningful features for distinguishing between acute sleep deprivation and well-rested wakefulness, we computed four complementary feature sets for each 20-second epoch. Time–frequency characteristics were obtained via a Continuous Wavelet Transform (CWT) (Büssow, 2007) using the Morlet wavelet, sampled at 256 Hz over a frequency range of 0.5–45 Hz (10 voices per octave), from which we derived amplitude and bandwidth modulation, spectral entropy, frequency centroid, peak amplitude and frequency, as well as skewness, kurtosis, and Hjorth mobility and complexity within the delta (0.5–4 Hz), theta (4–8 Hz), alpha (8–12 Hz), and beta (12–30 Hz) bands.

Power-spectral features were estimated using Welch’s method (Welch, 1967) with Hamming windows tailored to each canonical band (*10* s (sec) for delta, *8* s for theta, *4* s for alpha, and *2* s for beta), with 50% overlap. From these spectra, we computed absolute and relative band powers, theta/alpha, delta/theta, and beta/alpha ratios, as well as spectral edge frequencies at the 50% and 95% cumulative-power thresholds.

Functional connectivity was quantified by bandpass filtering each epoch into the four canonical bands and computing both the phase-locking value (PLV) (Aydore et al., 2013) and coherence (Nunez et al., 1997) averaged across all unique channel pairs. Graph-theoretic metrics (mean node strength, weighted clustering coefficient, global efficiency, characteristic path length, and modularity) were then derived from the full weighted PLV adjacency matrices. All extracted features were concatenated across channels into a single high-dimensional vector per epoch, resulting in a final feature vector of size *2481*.

To train the machine learning models in a supervised manner, labels were generated for each epoch. The well-rested wakefulness epochs were labelled 0, and the acute sleep deprivation epochs were labelled 1, making this a binary classification problem.

### 2.4 Model training and evaluation

#### 2.4.1 Machine learning models

In this study, we evaluated model performance using two complementary cross-validation strategies to capture both within-subject and cross-subject generalizability. Four machine learning models - RF, XGBoost, LightGBM, and Support Vector Classifier (SVC), were trained and tested within a fully nested cross-validation framework to obtain unbiased performance estimates. In the epoch-level approach (without subject-level separation), the outer loop employed a standard 5-fold stratified split with random shuffling, ensuring balanced class representation in each fold. To additionally assess cross-subject generalizability and mitigate potential inflation of performance estimates due to within-subject data leakage, we implemented a subject-level separation approach using a fully nested 5-fold stratified group k-fold scheme, whereby all epochs from a given participant were confined to either the training or test partition in each outer fold, ensuring no overlap of participant data between model development and evaluation. For both the epoch-level and subject-level approaches, 80% of the data was allocated to training and 20% to testing in each fold.

For both the epoch-level and subject-level separation analyses, feature scaling to the 0-1 range and feature selection were performed exclusively within each outer training partition to prevent information leakage from the held-out outer test fold. The feature selection pipeline, adapted from (Baygin, 2025), consisted of two sequential steps: (i) a univariate $\chi^{2}$ filter (Liu and Setiono, 1995) retaining the top 50 features, and (ii) a multivariate embedding step using neighborhood component analysis (NCA) (Goldberger et al., 2004) to select an additional 50 features based on their contribution to class discrimination. This approach yielded 100 features in total, a number chosen to balance model complexity with overfitting risk while retaining sufficient discriminatory information.

Hyperparameter optimization was conducted entirely within the inner loop of the nested framework. For each outer training set, an inner three-fold stratified cross-validation was performed, during which a randomized search explored 25 candidate parameter configurations (Table 1). The configuration with the highest mean validation performance was selected, retrained on the full outer training set (restricted to the selected features), and subsequently evaluated on the corresponding held-out outer test fold. This fully integrated approach, combining feature selection, hyperparameter tuning, and unbiased evaluation, ensures robust and clinically relevant estimates of generalizability. The models were trained using the Scikit-learn (Pedregosa et al., 2011) library in Python (Van Rossum and Drake, 2009).

**TABLE 1:** Grid search parameters used for hyperparameter tuning of the four machine learning models - LightGBM (Light Gradient-Boosting Machine), RF (Random Forest), XGBoost (eXtreme Gradient Boosting), and SVC (Support Vector Classifier).

Model name	Grid parameters
LightGBM	num_leaves, learning_rate, n_estimators, feature_fraction, bagging_fraction, bagging_freq, boosting_type
Random Forest	n_estimators, max_depth, criterion, max_features, bootstrap
XGBoost	n_estimators, learning_rate, max_depth, subsample, colsample_bytree
SVC	C, kernel, gamma

#### 2.4.2 Deep learning models

In addition to the aforementioned machine learning classifiers, we implemented a set of deep learning architectures to directly learn discriminative representations from the multi-channel EEG time series. Unlike traditional models, which rely on handcrafted features, these architectures operate on minimally processed data and are capable of jointly learning temporal, spectral, and spatial patterns relevant to sleep deprivation. Specifically, we evaluated three complementary network types: CNN, LSTM, and Transformer. The input to all models consisted of preprocessed multi-channel EEG signals, as shown in Figure 1. Consistent with the ML analyses, the deep learning models were also evaluated using both the epoch-level and subject-level separation approaches within a fully nested cross-validation framework. For the subject-level separation analysis, we used a stratified group k-fold scheme to ensure that data from each participant were assigned entirely to either the training or test set within a given outer fold.

The proposed CNN architecture initially applies a one-dimensional (1D) depthwise temporal convolution independently to each channel, facilitating efficient extraction of channel-specific temporal features. This is followed by a 1D pointwise convolution to integrate information across channels. Subsequently, two residual blocks are employed, each consisting of 1D depthwise temporal and 1D pointwise convolutions, along with batch normalization, exponential linear unit (ELU) activation, and dropout regularization. This configuration enables deeper feature refinement while preserving temporal resolution. The network concludes with an adaptive average pooling layer, a dropout layer, and a fully connected output layer for binary classification, enabling the model to learn discriminative spatiotemporal representations for the detection of acute sleep deprivation.

The LSTM model was implemented with a hidden size of 128 units in a single recurrent layer, employing a bidirectional architecture to capture temporal dependencies in both forward and backward directions. To mitigate overfitting, a dropout rate of 0.3 was applied after the recurrent layer. The output from the final timestep was passed through a fully connected layer to produce a binary prediction indicating the presence or absence of acute sleep deprivation.

The proposed transformer model first projects each channel vector at a given time step into a 64-dimensional embedding space via a linear layer, followed by sinusoidal positional encoding to preserve temporal order. A learnable classification ([CLS]) token is prepended to the sequence to aggregate global context for the final prediction. The model consists of a stack of multi-head self-attention encoder layers, each containing a multi-head attention module, position-wise feed-forward network, residual connections, layer normalization, and dropout regularization. The output corresponding to the [CLS] token is passed through a layer-normalized feed-forward classification head to produce the binary decision of acute sleep deprivation versus well-rested wakefulness states.

The models were trained using the AdamW optimization algorithm, configured with a learning rate of $1\times 10^{-4}$ and a weight decay parameter of $1\times 10^{-4}$ . Binary Cross-Entropy with Logits Loss (BCEWithLogitsLoss) was employed as the objective function. Training was conducted with a mini-batch size of 64. All models were implemented in Python using the PyTorch framework (Paszke et al., 2019).

#### 2.4.3 Model evaluation

Model performance was evaluated using accuracy, precision, recall, F1-score, and the area under the Receiver Operating Characteristic (ROC) curve (AUC). To obtain these measures, a confusion matrix was first constructed, summarizing outcomes as true positives (TP: correctly identified positive instances), true negatives (TN: correctly identified negatives), false positives (FP: negatives incorrectly classified as positives), and false negatives (FN: positives incorrectly classified as negatives).

From the confusion matrix, accuracy (Equation 1) was computed as the overall proportion of correctly classified samples:

$$\text{Accuracy}=\frac{TP+TN}{TP+TN+FP+FN}$$ (1)

Precision (Equation 2a) and recall (Equation 2b), which quantify the reliability of positive predictions and the model’s sensitivity to actual positives, respectively, were defined as:

$$\text{Precision}=\frac{TP}{TP+FP}$$ (2a)

$$\text{Recall}=\frac{TP}{TP+FN}$$ (2b)

The F1-score (Equation 3), defined as the harmonic mean of precision and recall, reflects the trade-off between these two measures, with higher values indicating better predictive capability:

$$\text{F}1-\text{Score}=\frac{2\cdot \text{Precision}\cdot \text{Recall}}{\text{Precision}+\text{Recall}}$$ (3)

The ROC curve was generated by systematically varying the decision threshold and, at each value, plotting the true positive rate (TPR (Equation 4a), equivalent to recall) against the false positive rate (FPR (Equation 4b), representing the proportion of negative instances incorrectly classified as positive).

$$TPR=\frac{TP}{TP+FN}$$ (4a)

$$FPR=\frac{FP}{FP+TN}$$ (4b)

The AUC was then calculated as the area under the ROC curve to provide a threshold-independent measure of the model’s discriminative ability, with values approaching 1 indicating superior performance.

### 2.5 Statistical analysis

To evaluate whether the observed differences in model performance were statistically significant, performance metrics derived from five-fold cross-validation were first subjected to the Friedman test (Friedman, 1937), a nonparametric alternative to repeated-measures analysis of variance (RM-ANOVA) (Girden, 1992). Analyses were conducted separately for each metric - accuracy, F1-score, and AUC. When the Friedman test indicated a significant overall effect, pairwise post hoc comparisons were carried out using Dunn’s test (Dunn, 1964) with multiplicity-adjusted p-values calculated to account for multiple pairwise comparisons.

### 2.6 Interpretability

Feature importance was quantified using Shapley Additive exPlanations (SHAP) (Lundberg and Lee, 2017), a game-theoretic approach that attributes the predictive output of a model to individual feature contributions. For each trained classifier, SHAP values were computed on the held-out test data of each outer cross-validation fold to ensure unbiased estimates. For non-tree-based models, feature attributions were computed using a model-agnostic SHAP framework with a representative subset of the training data serving as the background distribution. For tree-based models, including RF, XGBoost, and LightGBM, a tree-specific SHAP formulation was applied (Lundberg et al., 2018), with outputs expressed in terms of predicted probabilities for the positive class to ensure that the magnitude and scale of SHAP values were directly comparable across models. In all cases, background data were subsampled (maximum 200 instances) to improve computational efficiency while maintaining distributional representativeness. For each fold, feature selection was performed prior to SHAP computation to ensure that only the subset of features used by the model was evaluated. The resulting SHAP values, which represent the marginal contribution of each feature to the model output, were aligned across folds to the intersection of selected features. To obtain global feature importance, we calculated the mean absolute SHAP value for each feature across all samples and folds, providing a consistent ranking of features according to their overall predictive contribution.

## 3 Results

Table 2 summarizes the classification performance of the seven evaluated models in terms of mean accuracy, F1-score, and AUC across five test folds, reported as mean ± standard deviation. Results are presented separately for evaluations conducted without and with subject-level separation.

**TABLE 2:** Performance metrics for the seven classifiers - Light Gradient-Boosting Machine (LightGBM), Random Forest (RF), eXtreme Gradient Boosting (XGBoost), Support Vector Classifier (SVC), Long Short-Term Memory (LSTM), Convolutional Neural Network (CNN), and Transformer. The table is divided into two panels: the upper panel presents results obtained without subject-level separation, while the lower panel reports results with subject-level separation. For each model, the values represent the mean accuracy, F1-score, and area under the receiver operating characteristic curve (AUC) across five test folds, accompanied by the standard deviation (mean ± SD). All metrics range from 0 to 1.

Models	Without subject-level separation		
	Accuracy ± SD	F1-score ± SD	AUC ± SD
LightGBM	0.9483 ± 0.0101	0.9476 ± 0.0095	0.9892 ± 0.0055
Random Forest	0.9453 ± 0.0092	0.9441 ± 0.0096	0.9870 ± 0.0060
XGBoost	0.9542 ± 0.0151	0.9536 ± 0.0150	0.9862 ± 0.0081
SVC	0.8525 ± 0.0217	0.8480 ± 0.0223	0.9261 ± 0.0148
CNN	**0.9572 ± 0.0134**	**0.9558 ± 0.0148**	**0.9923 ± 0.0039**
LSTM	0.6675 ± 0.0262	0.6546 ± 0.0277	0.7289 ± 0.0281
Transformer	0.7739 ± 0.0745	0.7944 ± 0.0589	0.8728 ± 0.0508

	With Subject-Level Separation		
LightGBM	0.6621 ± 0.0352	0.6566 ± 0.0396	0.7147 ± 0.0399
Random Forest	**0.6823 ± 0.0217**	**0.6702 ± 0.0451**	**0.7290 ± 0.0391**
XGBoost	0.6636 ± 0.0274	0.6533 ± 0.0395	0.7053 ± 0.0335
SVC	0.6508 ± 0.0572	0.6276 ± 0.1068	0.6951 ± 0.0630
CNN	0.6635 ± 0.0529	0.6415 ± 0.0806	0.7196 ± 0.0477
LSTM	0.6170 ± 0.0320	0.6059 ± 0.0455	0.6662 ± 0.0461
Transformer	0.6335 ± 0.0278	0.6367 ± 0.0431	0.6981 ± 0.0529

Bold values represent the best performing models.

In the evaluation without subject-level separation, CNN achieved the highest mean accuracy (0.9572 ± 0.0134), F1-score (0.9558 ± 0.0148), and AUC (0.9923 ± 0.0039). XGBoost followed closely, with an accuracy of 0.9542 ± 0.0151, F1-score of 0.9536 ± 0.0150, and AUC of 0.9862 ± 0.0081. LightGBM and Random Forest exhibited comparable performance, with accuracies of 0.9483 ± 0.0101 and 0.9453 ± 0.0092, respectively. SVC attained an accuracy of 0.8525 ± 0.0217, F1-score of 0.8480 ± 0.0223, and AUC of 0.9261 ± 0.0148. The Transformer yielded an accuracy of 0.7739 ± 0.0745, F1-score of 0.7944 ± 0.0589, and AUC of 0.8728 ± 0.0508, while LSTM recorded the lowest performance among all models in this setting, with an accuracy of 0.6675 ± 0.0262, F1-score of 0.6546 ± 0.0277, and AUC of 0.7289 ± 0.0281.

**FIGURE 2:**
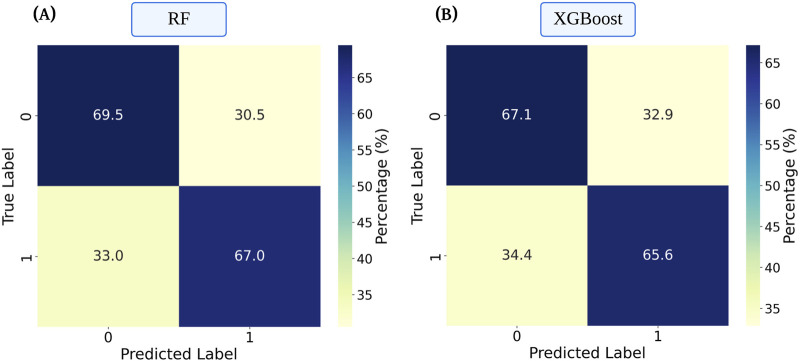
Confusion matrices for the two best-performing models with subject-level separation - **(A)** Random Forest (RF), **(B)** eXtreme Gradient Boosting (XGBoost). The label 0 refers to well-rested wakefulness, and 1 refers to acute sleep deprivation. A probability cutoff of 0.5 was used to derive binary classifications. Values in the matrices are expressed as row-normalized percentages, indicating the proportion of samples within each true class assigned to each predicted class.

With subject-level separation, performance values were lower for all models. RF classifier obtained the highest mean accuracy (0.6823 ± 0.0217), F1-score (0.6702 ± 0.0451) and AUC (0.7290 ± 0.0391). LightGBM and XGBoost yielded similar results, with accuracies of 0.6621 ± 0.0352 and 0.6636 ± 0.0274, respectively. CNN achieved an accuracy of 0.6635 ± 0.0529, F1-score of 0.6415 ± 0.0806, and AUC of 0.7196 ± 0.0477. SVC recorded an accuracy of 0.6508 ± 0.0572, F1-score of 0.6276 ± 0.1068, and AUC of 0.6951 ± 0.0630. The transformer model yielded an accuracy of 0.6335 ± 0.0278, an F1-score of 0.6367 ± 0.0431 and an AUC of 0.6981 ± 0.0529, while LSTM reported the lowest accuracy of 0.6170 ± 0.0320, F1-score of 0.6059 ± 0.0455, and AUC of 0.6662 ± 0.0461. Confusion matrices for the two best-performing models with subject-level separation are presented in Figure 2.

**FIGURE 3:**
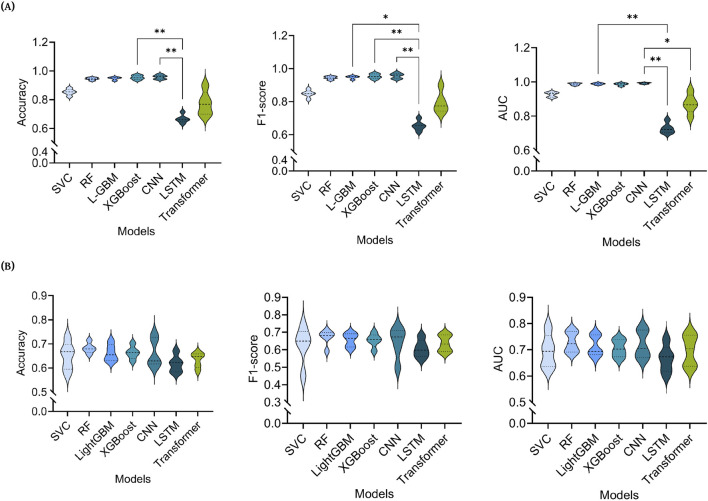
Statistical Analysis. A Friedman test was performed to evaluate the statistical significance of observed differences in performance, for each evaluation metric, across all models - SVC (Support Vector Classifier), RF (Random Forest), LightGBM (Light Gradient-Boosting Machine), XGBoost (eXtreme Gradient Boosting), CNN (Convolutional Neural Network), Long Short-Term Memory (LSTM) and a transformer-based model. Dunn’s test was applied for multiple pairwise model comparisons. The results of the statistical analysis for **(A)** without subject-level separation and **(B)** with subject-level separation, across the three metrics - Accuracy, F1-score, and Area Under the Curve (AUC) - are presented.

At the epoch level (Figure 3A), Friedman tests revealed significant overall differences in model performance for accuracy (p = 0.0003), F1-score (p = 0.0003), and AUC (p = 0.0002). Post hoc Dunn’s tests with multiplicity-adjusted p-values showed that, for accuracy, the LSTM model performed significantly worse than both XGBoost (rank-sum difference = +25.0, p = 0.0053) and CNN (+24.5, p = 0.0070). For F1-score, LSTM again underperformed relative to LightGBM (+22.0, p = 0.0269), XGBoost (+24.0, p = 0.0093), and CNN (+25.0, p = 0.0053). For AUC, LSTM achieved significantly lower scores than LightGBM (+24.0, p = 0.0093) and CNN (+27.0, p = 0.0016), while CNN also outperformed the Transformer (+21.0, p = 0.0443). No other pairwise comparisons reached statistical significance after correction. Here, the rank-sum difference represents the difference between the sums of the within-fold ranks assigned to each model in the Friedman procedure, with positive values indicating that the first-listed model achieved higher (better) ranks.

With subject-level separation (Figure 3B), Friedman tests showed no overall differences among model performance (accuracy: p = 0.0823, F1-score: p = 0.4137, AUC: p = 0.7076). Consistent with this, Dunn’s multiplicity-adjusted pairwise comparisons found no significant contrasts. A modest trend was noted for accuracy, with RF ranking above LSTM (rank-sum difference = +20.0, adjusted p = 0.0717). All other adjusted p-values were ≥0.40 .

**FIGURE 4:**
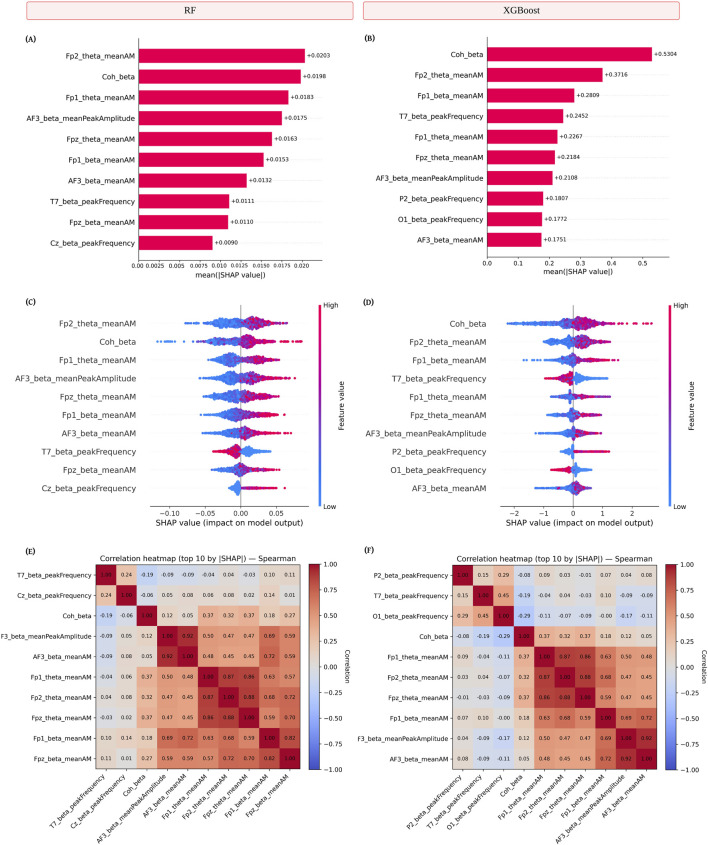
Feature importance using Shapley Additive exPlanations (SHAP) for the two best-performing models with subject-level separation–Random Forest (RF) (Left) and eXtreme Gradient Boosting (XGBoost) (Right). **(A,B)** Mean absolute SHAP values for the top 10 features, indicating average contribution magnitude to model predictions. **(C,D)** SHAP beeswarm plots showing feature impact distributions, with color denoting normalized feature values (blue = low, red = high). **(E,F)** Spearman correlation heatmaps for the top 10 SHAP-ranked features, illustrating inter-feature associations and potential collinearity. Features are listed in the format {ChannelName}_{FrequencyBand}_{FeatureName} . Here, *meanAM* refers to the mean amplitude modulation and *coh_beta* refers to the coherence in the beta band.

SHAP analysis was conducted to identify the most influential features for the two best-performing models under subject-level separation: RF and XGBoost. In the RF model, the highest mean absolute SHAP value was observed for *Fp2_theta_meanAM* (mean amplitude modulation in the theta band at the right frontal site), followed by *Coh_beta* (average magnitude-squared coherence between all channel pairs in the beta band), *Fp1_theta_meanAM* (mean amplitude modulation in the theta band at the left frontal site), and *AF3_beta_meanPeakAmplitude* (mean peak amplitude within the beta band, indicating the strength of the strongest oscillations) (Figure 4A). The XGBoost model ranked *Coh_beta* as the most significant feature, followed by *Fp2_theta_meanAM*, *Fp1_beta_meanAM*, and *T7_beta_peakFrequency* (single frequency within the beta band at the left temporal site T7 with the highest total energy over the epoch) (Figure 4B). While both models identified *Fp2_theta_meanAM* and *Coh_beta* among their top contributors, the differences in ranking suggest that each algorithm prioritizes distinct but partially overlapping neural markers.

Beeswarm plots (Figures 4C,D) illustrated both the magnitude and directionality of each feature’s influence on the predicted class. Higher *Fp2_theta_meanAM* values consistently increased the likelihood of a sleep deprivation prediction in both models, indicating that elevated frontal theta modulation is a key discriminator. In contrast, *Coh_beta* exhibited a more variable influence, with both high and low values contributing in different directions, suggesting interaction effects with other features.

Correlation heatmaps (Figures 4E,F) indicated moderate-to-strong positive correlations among spectral features within neighboring frontal sites. In the RF model, *Fp1_theta_meanAM* and *Fp2_theta_meanAM* were strongly correlated. In the XGBoost model, a particularly high correlation was observed between *Fpz_theta_meanAM* (midline frontal theta modulation) and *Fp2_theta_meanAM*, suggesting shared frontal theta activity across midline and lateral sites. Across both models, *Coh_beta* showed relatively low correlations with amplitude or frequency-based features, highlighting its distinct connectivity-related contribution.

In summary, both models relied heavily on frontal theta-band amplitude modulation and beta-band connectivity, underscoring the complementary roles of spectral dynamics and large-scale functional coupling in distinguishing sleep-deprived from well-rested states.

## 4 Discussion

In this study, using an open-source eyes-open resting-state EEG dataset, we investigated the feasibility of detecting acute sleep deprivation with machine learning models. We initially report performance at the epoch level, where data from the same participant may be present in both training and test sets. While this evaluation yielded notably high accuracies, particularly for the CNN, these results are likely inflated due to subject-specific data leakage and temporal dependencies. To address these limitations and assess real-world generalizability, we conducted a more rigorous subject-level evaluation, ensuring that no participant contributed data to both training and test sets. Under this more realistic setting, performance declined across all models, underscoring the difficulty of generalizing to previously unseen individuals. Notably, the RF classifier achieved the highest accuracy in this configuration and demonstrated greater robustness to inter-subject variability compared to the other models. These findings highlight the importance of subject-level validation in the development of EEG-based sleep deprivation detection systems.

The superior performance of RF under subject-level separation may be attributed to the tendency of deep learning models to overfit when trained on relatively small datasets, as in the present study. Deep architectures, while powerful in capturing complex nonlinear patterns, require large volumes of diverse training data to generalize effectively. In contrast, traditional ensemble-based methods such as RF and XGBoost are less susceptible to overfitting in low-data regimes due to their inherent regularization mechanisms, bootstrap aggregation, and feature subspace sampling. These properties enable them to maintain more stable performance when confronted with limited training examples. We hypothesize, however, that with access to substantially larger and more diverse acute sleep deprivation-related EEG datasets, deep learning models may surpass traditional approaches in both accuracy and generalizability.

Feature importance derived from SHAP analysis revealed that the most influential predictors for the best-performing models (with subject-level separation) aligned closely with established neurophysiological effects of acute sleep deprivation (Figure 4). Neurophysiologically, sleep deprivation is associated with increased slow-wave activity, particularly in the theta band (4–7 Hz), reflecting drowsiness and the transition from beta to theta rhythms during the onset of sleep (Iber et al., 2007). Our analysis identified frontal theta-band amplitude modulation, most prominently at *Fp2*, *Fp1*, and *Fpz*, as consistently important across both Random Forest and XGBoost models, in line with evidence that frontal theta activity increases during sustained wakefulness, accompanied by significantly higher subjective sleepiness and decreased alertness (De Gennaro et al., 2007). In addition, beta-band connectivity (*Coh_beta*) emerged as a key contributor, representing large-scale functional coupling between cortical regions within the 12–30 Hz range. Intermittent increases in beta activity during sleep deprivation have been linked to micro-awakenings or brief periods of heightened cortical activation, potentially reflecting compensatory mechanisms to maintain alertness (Craig et al., 2012). The identification of beta-band peak amplitude and peak frequency features, particularly in frontal and temporal regions such as *AF3* and *T7*, further supports the view that oscillatory dynamics in these bands play complementary roles in distinguishing between well-rested and sleep-deprived states. However, contrary to earlier findings indicating elevated delta-band power as a marker of sleep deprivation (De Gennaro et al., 2007), our models did not identify delta-related features among the top predictors. One possible explanation is methodological: the use of 20-second epochs, while stabilizing spectral estimates, may reduce sensitivity to transient delta fluctuations. In addition, substantial inter-subject variability in delta power likely diminishes its discriminative value for multivariate classification. These biological and methodological considerations may account for why delta features did not emerge as dominant predictors in our study. Collectively, the SHAP results suggest that the discriminative features leveraged by the ensemble models are neurophysiologically meaningful, capturing both the spectral slowing characteristic of drowsiness and the transient beta synchrony associated with compensatory arousal mechanisms.

Focusing on studies that have utilized machine learning for the classification of acute sleep deprivation, a novel feature extraction method called MelPat was introduced in (Baygin, 2025) and applied to the same open-source EEG dataset used in this study, achieving 97% accuracy with an SVC classifier. While this approach demonstrated high performance, our study in contrast, employed a traditional, well-established EEG feature set that achieved comparable accuracy while enabling us to link discriminative features to established neurophysiological biomarkers, thereby enhancing interpretability. Furthermore, unlike MelPat, we also evaluated model performance under a subject-level separation scheme, providing a more rigorous assessment of cross-subject generalizability.

Our work offers several notable contributions. First, unlike prior studies (Baygin, 2025) in this domain, we explicitly evaluated both epoch-level and subject-level separation schemes, with the latter providing a more realistic estimate of model performance in real-world scenarios by preventing within-subject data leakage. Second, we assessed a broad spectrum of approaches, including both traditional machine learning models and deep learning architectures, enabling a comprehensive comparison of their relative strengths. While deep learning models demonstrated strong performance under the less stringent epoch-level setting, our analysis also highlighted their vulnerability to overfitting in limited-data contexts, underscoring the need for larger and more diverse datasets to realize their full potential. Third, our study placed strong emphasis on model interpretability by employing SHAP analysis, which offers a unified, model-agnostic framework for quantifying the contribution of individual features to model predictions. This approach not only facilitates transparency but also enables direct correspondence between the most influential features and established neurophysiological markers of sleep deprivation, thereby enhancing the clinical relevance and applicability of our findings.

This study has certain limitations that should be considered when interpreting the findings. The analysis is based on an open-source eyes-open resting-state EEG dataset obtained from a demographically homogeneous cohort of young, healthy adults, which may restrict the generalizability of the results to more diverse populations. Replication and benchmarking against independent datasets with comparable experimental protocols would enhance the robustness of our conclusions. However, this was not feasible due to the lack of suitable publicly available datasets. The present work conceptualized acute sleep deprivation as a binary state, whereas its neurocognitive and physiological effects likely manifest along a graded continuum. This simplification may obscure more nuanced patterns of impairment and recovery, and represents an inherent limitation of the current design. In the absence of a provided reference channel in the open-source dataset, we employed a common average reference (CAR). However, CAR can be sensitive to montage density and scalp coverage and may influence sensor-level topographies and connectivity (Yao et al., 2019). As we did not formally compare alternative re-referencing schemes, residual reference-related distortions cannot be excluded. While the use of non-overlapping 20-second epochs was chosen to balance temporal resolution with the stability of spectral estimates, this segmentation approach may limit sensitivity to transient or rapidly evolving neural dynamics.

## 5 Conclusion and future work

This study represents an important step toward the development of EEG-based machine learning models for the objective detection of acute sleep deprivation and the characterization of its impact on cognitive readiness. While the best-performing model in this study achieved an accuracy of 68%, with subject-level separation, these findings demonstrate the overall viability of EEG-driven approaches for monitoring sleep deprivation in operational contexts. However, real-world deployment will require models with significantly higher performance, trained on larger and more diverse datasets. The present work lays the essential groundwork for such advancements by establishing a robust and interpretable pipeline that captures meaningful neurophysiological signatures of acute sleep loss. It is also important to recognize that the observed effects reflect transient, state-level changes associated with acute sleep deprivation, rather than stable, trait-like characteristics of individuals. Future research should examine how individual variability and longitudinal trajectories influence susceptibility to, and recovery from, acute sleep deprivation, thereby extending the present findings beyond transient state-level effects.

Future research should also consider adopting multi-class or regression-based frameworks to better capture the graded, impact of sleep deprivation on cognitive and physiological function. Expanding model inputs to include demographic variables such as age and sex will also be essential to improve generalizability across populations. Furthermore, exploring advanced EEG features, such as dynamic functional connectivity (dFC) (Chen et al., 2024) and microstate analysis (Khoo et al., 2024), may offer complementary insights that improve classification performance while deepening our understanding of the underlying neural mechanisms. Future work can also explore multi-scale or overlapping windowing strategies to better capture fine-grained temporal dynamics. Finally, integrating more accessible physiological modalities such as photoplethysmography (PPG) may help translate these models into real-world, wearable monitoring systems, ultimately supporting continuous, non-invasive assessment of sleep-related cognitive readiness.

## Data Availability

Publicly available datasets were analyzed in this study. This data can be found here: OpenNeuro repository (Dataset: DS004902, version: 1.0.8): https://openneuro.org/datasets/ds004902/versions/1.0.8 (doi: 10.18112/openneuro.ds004902.v1.0.8).
